# Quality of health information for cervical cancer treatment on the internet

**DOI:** 10.1186/1472-6874-6-9

**Published:** 2006-06-20

**Authors:** Tara J Selman, Trina Prakash, Khalid S Khan

**Affiliations:** 1 Academic Department, Birmingham Women's Hospital, Birmingham, UK

## Abstract

**Background:**

The internet has become a frequently used and powerful tool for patients seeking medical information. This information may not undergo the same quality consideration as the peer-review criteria for publication of information in a journal. The aim of this study is to assess the quality of internet sites providing information on the treatment of cervical cancer, with comparisons between the quality assessments made by an educated lay person and an expert in the field.

**Methods:**

A search of the World Wide Web was made by a lay person to identify sites containing information on the treatment of cervical cancer. The credibility and accuracy of these sites was assessed using predefined criteria based on 'Criteria for Assessing the Quality of Health Information on the Internet' and accepted guidelines for the treatment of cervical cancer. The assessment was made independently and in duplicate by the lay reviewer and medical expert in order to allow comparison.

**Results:**

46 relevant websites were assessed. Only one site contained all the credibility and accuracy criteria, with a further website containing all the credibility criteria. The majority of sites, 38/46, were deemed easy to navigate. The agreement between lay person and expert was good with only 6 items in total changed by the expert.

**Conclusion:**

This study clearly shows there is wide variation in quality of websites available to patients on the treatment of cervical cancer. Further research and consideration is needed on the effects of website information on gynaecological cancer patients and how steps can be made to insure the posting of good quality information.

## Background

The internet has become a frequently used and powerful tool for patients seeking medical information. A Harris Poll showed that between 1998–1999 more than 70 million adults used the internet to find health information, a number that was predicated to be rapidly increasing each month [1]. The number of health related web sites is rising in line with the demand, with more than 70000 sites available to patients in 2000 [1]. Not only do patients access this information, but it seems that it is influencing there treatment choice. In the United States more than 70% of patients reported the health information they found influenced there treatment decision [2].

Undoubtedly for cancer patients, as for all patients, the internet can be an excellent tool for reinforcing the information given by health care providers, further supplementing knowledge and providing a useful medium to prompt additional questions. Unfortunately, despite these advantages, unsolicited posting of web sites can result in as much harm as good. To help avoid the potential harm from such sites it is essential that information provided by the internet undergoes quality consideration in a way similar to peer-review criteria for publication of information in a journal. Unlike the majority of journals it is the patient, rather than the health care provider that is accessing this information directly. It may not be feasible or appropriate to transfer the responsibly of critiquing such information to patient support groups. Ultimately no suitable method exists for the policing medical web site.

Guidelines have been development for the quality assessment of websites [3,4] in a similar way to the guidelines for journal manuscripts. There are, however, many additional difficulties in regulating quality control for the internet. These range from problems maintaining validity of a rapidly changing, dynamic source to problems taking account of the potentially wide variety of user needs [5]. To date we are unaware of any studies that have attempted to examine the accuracy of websites information related to gynaecological cancer. In our practice it is apparent that our patients with cervical cancer are turning to the internet for information on their treatment options. Cervical cancer effects approximately 2500 women per year in England [6] and effects an increasing cohort of younger women for whom internet is a familiar medium to access information. This study aimed to assess the quality of internet sites providing information on the treatment of cervical cancer, with comparisons made between the quality assessment made by an educated lay person and an expert in the field.

## Methods

### Identification of websites

This study was carried out from the perspective of a lay women diagnosed with cervical cancer. A questionnaire survey was used to ascertain the internet search strategy of a lay person who would potentially access internet information on cervical cancer treatment. Twenty women between the ages of 25 to 50 years were questioned on the search engines used and an estimate of the number of citations checked to guide the development of our own search strategy. The search engine Copernick was used to search the web. This allowed efficient searching of the web as it uses the most commonly cited search engines and deletes duplicates. The first two pages of Google and Ask Jeeves were also searched separately to capture any additional web sites. The phrase 'treatment of cervical cancer' was entered as the search term and all web pages from Copernick were downloaded. The Websites down loaded were assessed in duplicate (TP and TJS). Websites were excluded if they were found to not actually be relevant, or if the option of translation to English was not available.

### Assessment of Quality

Much debate still remains surrounding the methods that should be used to evaluate the quality of website information and which methods are most appropriate [4,5]. In the absence of a consensus on an appropriate, validated tool our quality assessment was based on Criteria for Assessing the Quality of Health Information on the Internet [3] with consideration of other quality checklists [7,8].

Quality can be defined as "the totality of characteristics of an entity that bear on its ability to satisfy stated and implied needs"[5], we used two main headings to asses the quality of the internet sites: credibility and accuracy of information.

The assessment of credibility was made by examining the following factors pertaining to a website: The source, with trusted authorities presumed to provide higher quality sites, we felt it acceptable if the source was stated; the currency, with currency being the data of the posting the document and any updates, which is extremely important as medical treatments continually evolve, it was deemed adequate if this was stated; the relevance, assessing if actual content of the site answers the search question posed; the stated use of an editorial process. The medical content of the web site was assessed for its accuracy. In order to asses this a list of fundamental treatment options was compiled from those recommended by FIGO [9] and national guidelines [10]. It was considered imperative that a site should comment on the staging of disease (the necessity for staging to be performed), the treatment of disease by stage, the necessary follow up (it was deemed sufficient to state hospital follow up was requires) and the treatment for recurrence (the potential for chemo or radiotherapy). We also recorded if the site provided journal references or an indication of the level of evidence for its information which is necessary in assessing the validity of the content, although we did not make any formal assessment of the quality of references.

In order that a patient can decide if the site is designed and specifically relevant for their needs the presence of a disclaimer was reordered. A subject assessment of the design of a site was made by the lay person, giving an opinion on the ease of navigating the site and acquiring the desired information. Finally an assessment of interactivity was made, which for the purposes of this study we limited to reporting if there was a mechanism for site feed back.

### Data extraction and analysis

A data extraction formed was designed to record the information outlined above. This provided a checklist of items for reviewers to ascertain if present and in this way make an assessment of accuracy. Data was extracted from each web site by a lay person (TP) and repeated independently by a research fellow in Obstetrics and Gynaecology (TJS). The lay reviewer had minimal prior knowledge of the subject. For each of the quality items the total number of websites containing these items was recorded as a percentage of the total number of sites.

## Results

### Web Search

**Figure 1:**
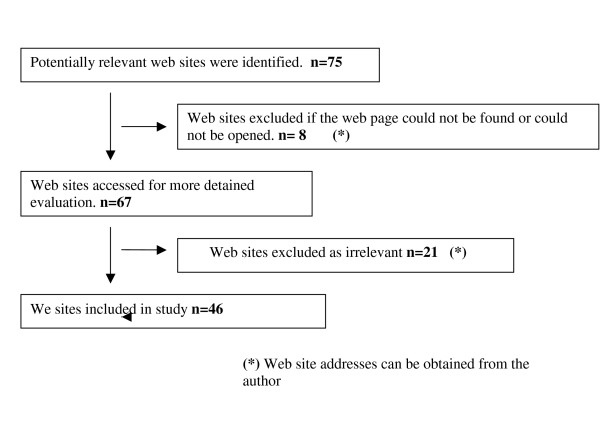
The selection process for relevant health web sites for the treatment of cervical cancer.

The initial web search found 75 web sites, of these 8 could not be accessed either due to the need for registration or a password, or unavailability on line. From the remaining 67 websites we deemed 46 to be relevant to answer our question (figure 1). These websites were accessed and assessed for their quality as previously described.

### Quality Assessment

**Figure 2:**
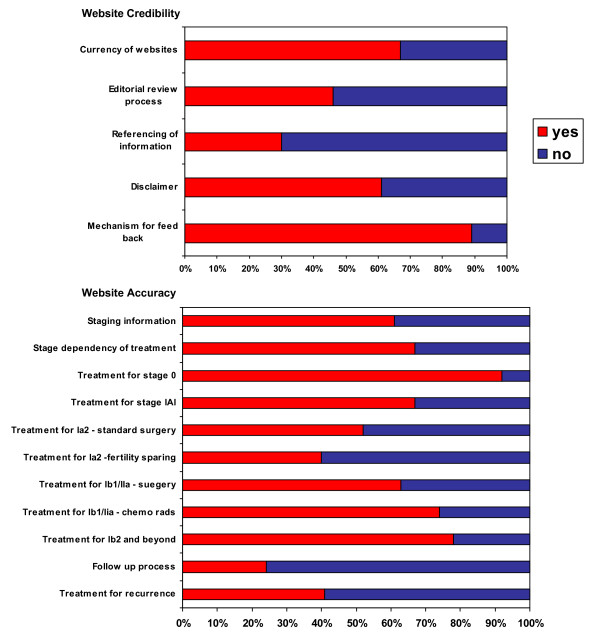
The proportion of websites displaying predefined quality criteria.

Figure 2 summaries the quality of the 46 accepted websites. There was only one site that included all website credibility and accuracy criteria [11]. In the assessment of the website credibility alone a further website included all our deemed essential factors [12]. The currency of the website was reported in 31/46 sites. An editorial process was evident in 21/46 websites. The information posted on a websites was referenced in only 14/46 sites, with a disclaimer present on 28/46 websites. The most frequently occurring credibility point present in the websites was a mechanism for feed back present in 41/46 sited.

In the assessment of the websites accuracy component of quality we did not identify any other websites that included all data we felt should be available; however nine websites did include 10 out of the 11 points. Information on the follow up necessary or the treatment of recurrent disease were the most frequently excluded points, they were identified in 11/46 and 19/46 websites respectively. The process of staging was reported in 28/46 websites, with 31/46 commenting on the treatment choice being stage dependant. Treatment for stage 0 disease was correctly posted in 33/46 sites and for stage IaI in 32/67. For stage Ia2, 27/46 websites posted information on routine surgical treatment and a further 17/46 websites posted information on alternative fertility sparing treatment. For the later stages of disease 29/46 websites posted information on surgical treatment and 34/46 websites posted information on chemo radiotherapy treatment. Finally 36/46 sites had correct information on the treatment of advanced disease.

An assessment was made by our non medical reviewer (TP) as to the ease with which the website could be navigated and the necessary information extracted. 38/46 websites were deemed reasonably easy to use.

## Discussion

This study reveals that there are a large number of easily accessible websites proving information on the treatment of cervical cancer, however there is much variation in quality of these sites. Worryingly and in line with previous papers in other areas there are major downfalls in the credibility of websites with only 1/46 websites posting all of the credibility points. The accuracy of information was not as weak in comparison, but there were still major gaps in the information posted with the treatment of stage 0 disease being the only option correctly reported in over 90% of the websites. The information extracted by our non medical reviewer was changed by the medical expert in 6/46 websites and the chance corrected agreement between reviewers for the various items of data extractionranged fromkappa statisticof 0.98 to 1.0. In all but one case the information changed related to extracting information on the treatment options for varying stages of disease, suggesting that there were deficiencies in a websites ability to publish information suitable for a lay person.

Our assessment of both the credibility and accuracy displayed by the websites and hence our overall assessment of quality does depend on the criteria we used in our assessment. There have been other quality factors which authors have considered, but our selection was made on those that would be most relevant to a medical health site. Our website search was made by a lay person with out any extra computer training in order to best mimic those women that might search for information on cervical cancer treatment, although this does make the assumption that one persons experience is representative of the population as a whole. This does however mean that it is possible that not all sites were identified. Despite this we feel that the results are representative of those that might be found by a patient.

It is indisputable that there is an ever increasing use of the internet by our patients. This study highlights the potential pitfalls facing a patient when accessing information on the treatment of cervical cancer and the necessity for health care professionals to ensure that patients are guided to the best quality internet sites and that their treatment pathways are explained to them in full. Our lay person was able to extract, without correction, all credibility points which may suggest that it is possible to transfer some of the responsibility of assess quality of information onto the public if suitable guidelines were available. The problem of extraction data on the treatment options for cervical cancer highlights previous concerns that websites posted have many different target audiences and some site may not have been aimed at a lay person level, it is therefore important that sites should make it obvious there intended target audience and that people consider inclusion of lay people when designing a web site for non medical targets [13].

## Conclusion

This study demonstrates the urgent need for further research and consideration on the effects of website information on gynaecological cancer patients and how steps can be made to insure the posting of good quality information.

## Competing interests

The author(s) declare that they have no competing interests.

## Authors' contributions

TJS was involved in the conception and design, performed a review of the results drafted the manuscript.

TP carried out the data extraction and participated in drafting the manuscript.

KSS conceived and coordinated the study and participated in drafting the manuscript.

All authors have read and approved the final manuscript.

## Pre-publication history

The pre-publication history for this paper can be accessed here:


